# The Story of the Insane from Year to Year

**Published:** 1904-10-08

**Authors:** 


### THE STORY OF THE INSANE FROM YEAR TO YEAR.

SEVENTEENTH ANNUAL SERIES.

### MULLINGAR DISTRICT ASYLUM.

THIS institution has accommodation for 864 patients, and at the end of the year 858 were in residence, so that it is practically full, although a block for 146 working patients was lately opened. This has relieved the male wards, but no additional room has been provided on the female side, and it is much overcrowded. We are told that the female staff is "also crowded together into dormitories to such a degree as to deprive the occupants of all comfort and privacy." Someone is responsible for this state of affairs, and we should like to ask why it has not been remedied. The duties of nurses for the insane are of the most trying description, and everything reasonable ought to be done to make their own surroundings cheerful and their hours off duty as pleasant as possible. Every nurse ought to have a bedroom to herself. There were 116 first admissions, 28 re-admissions, 58 discharged recovered, 9 discharged relieved, 2 discharged not improved, and 67 died. The total number under treatment during the year was 998, and the average number resident was 860. The recoveries were 40·27 per cent. of the admissions, and the deaths were 7·79 on the average number resident. The first of these percentages is quite up to or above the average and the second is considerably below it. As to the causes of insanity in the year's admissions, we find that only 6 cases were attributed to moral causes; and among the physical causes only 7 to drink, while hereditary tendency accounted for 44, previous attacks for 17, and congenital defect for 10. In 32 there were no causes known. Of the year's deaths 13 were due to cerebral and spinal diseases (general paralysis only appearing once in the returns), 15 to consumption, and 9 to other lung diseases. Every asylum ought to have a detached hospital for infectious diseases; but here there is none, and it is not surprising to learn that scarlatina has been almost endemic lately. The staff of attendants is in the proportion of 1 to 14 patients, and that of the nurses is 1 to 15 patients. This is decidedly too low even if not lessened by illness and absence, which it is sure to be occasionally, and the smallness is even more to be regretted when it is pointed out that some of the nurses are below the standard height and not physically suited for the work. No wonder that the escapes of patients were numerous. Between October 1st and December 11th 14 were able to get away. We could find no report by the chairman of the committee and no report by the medical superintendent. The auditor states that "the committee do not appear to examine the subsidiary accounts before making payments, as required under Articles 6 and 9 of the Asylum Accounts Order, 1899," and further that, "the clerk does not check the storekeeper's books." There are only two assistant medical officers for 860 patients. The average cost per head per annum is £24 2s.

